# Postgraduate Psychiatry Training Programme in Morocco

**DOI:** 10.1192/bjo.2022.134

**Published:** 2022-06-20

**Authors:** Imane Salihi, Jiann Lin Loo

**Affiliations:** 1 Casablanca University Hospital, Casablanca, Morocco; 2 Betsi Cadwaladr University Health Board, Wrexham, United Kingdom

## Abstract

**Aims:**

The field of psychiatry in Morocco has grown significantly since the 1970s, from less than 10 psychiatrists to the current number of around 400. The increased number of practising psychiatrists has enabled the expansion of local residency training programmes, which has been set up since 1974 to cater for the population needs of more than 36 million population of Morocco. This study is aimed to describe the current medical educational approach of the Moroccan postgraduate psychiatry training programme.

**Methods:**

This descriptive medical educational study was based on official training documents and interviews with local faculty members involved in the training.

**Results:**

The entry requirement of the four-year Moroccan postgraduate psychiatry residency programme includes the completion of 1 year of foundation training and passing the entrance examination consisting of psychiatric semiology and pharmacology. The postgraduate residency programme is run by the local universities in collaboration with the Ministry of Health and accredited by the Moroccan government. Trainees have the option of taking up a voluntary or contractual position with the government or University Hospitals. All trainees will go through 34 months of general adult outpatient and inpatient, while liaison psychiatry training starts from the second year until the end of the training. On top of the core rotation, a trainee can opt for two months in old age and neuropsychiatry postings. Child and adolescent rotation is currently not available. Addiction psychiatry training is optional and can be done through a university diploma. The 4th year is a 12-month elective posting in any discipline that is relevant to psychiatry, which can be done either locally or abroad. Teaching methodologies involve lectures, seminars, ward rounds, case conferences, journal clubs, and skill training workshops. Formative assessments included case-based discussions and mini-clinical evaluation exercise. There are multiple high stakes summative assessments at year 1, year 2, year 3, and year 4. The summative assessment strategies includes modified essay question, clinical short case and long case. Viva voce is used to assess competency in research. Different mandatory skill competencies include electroconvulsive therapy, psychotherapy, and research.

**Conclusion:**

The advancement of local postgraduate psychiatry residency training in Morocco has improved the access of local trainees to quality training. Similar to other developing countries, Morocco requires more psychiatrists to improve the psychiatrists to population ratio so that the mental service can become more accessible to the local population.

